# Joint latent profiles of workplace violence and burnout among healthcare workers and their associated factors: a cross-sectional study

**DOI:** 10.3389/fpsyt.2026.1922345

**Published:** 2026-09-21

**Authors:** Tao Duan, Xiaohong Zou, Lijuan Zeng, Jie Chen, Qiao Li, Cheng Chen

**Affiliations:** Information Center, Affiliated Hospital of North Sichuan Medical College, Nanchong, Sichuan, China

**Keywords:** burnout, emotional labor, healthcare workers, work-family conflict, workplace violence

## Abstract

**Objective:**

To identify the joint latent profiles of workplace violence and burnout among healthcare workers using a person-centered approach, and to examine the psychosocial and demographic factors associated with profile membership.

**Method:**

A cross-sectional survey was conducted among 616 healthcare workers recruited from six hospitals in Sichuan Province, China, between April and May 2026. Workplace violence, burnout, work-family conflict, and emotional labor were assessed using validated self-report questionnaires.

**Results:**

A two-class model provided the optimal solution. Class 1, the “relatively adaptive profile”, was characterized by low levels of workplace violence and burnout across all dimensions. Class 2, the “high-risk profile”, exhibited significantly elevated workplace violence and burnout, accompanied by a prominent level of work-family conflict; more than half of the participants belonged to this profile. In the multivariable model, work-family conflict was the strongest independent predictor of high-risk profile membership. Although gender, hospital role, educational level, and monthly income differed significantly between profiles in univariate analyses, none remained significant after adjustment.

**Conclusion:**

These findings suggest that occupational mental health interventions should prioritize workplace violence prevention, the promotion of work–family balance, and that such psychosocial intervention pathways may have applicability across demographic subgroups.

## 1 Introduction

Workplace violence has become a persistent and highly complex occupational health problem in healthcare systems worldwide (1, 2). Violent incidents in healthcare settings typically encompass overt forms of violence such as physical assault, threats, verbal abuse, and intimidation, as well as covert forms including cold violence, verbal humiliation, cyberattacks, and institutional disrespect (3, 4). Previous studies have found that workplace violence not only compromises the physical safety and mental health of healthcare workers (5, 6) but may also undermine their professional identity, organizational trust, and clinical decision-making confidence, thereby affecting the quality of healthcare services and patient safety (7, 8). This is largely because healthcare workers maintain close contact with patients and their families in high-pressure situations, rendering them more susceptible to tense doctor–patient interactions, conflicts over resource allocation, and emotional venting behaviors. Accordingly, workplace violence in healthcare can be understood as the product of interactions among multiple factors, including strained medical resources, communication barriers between providers and patients, declining social trust, and weak occupational risk management (9). Systematically identifying the factors influencing workplace violence among healthcare workers is therefore of considerable importance for developing stratified and precision-oriented occupational protection strategies.

Burnout is a state of chronic psychological exhaustion that develops in healthcare workers following prolonged exposure to high-intensity occupational stress (10, 11), characterized by emotional exhaustion, depersonalization, and a diminished sense of personal accomplishment (12). Because healthcare workers bear work tasks involving high responsibility, high risk, and high emotional load over the long term (13), often required to complete complex diagnosis and treatment, nursing procedures, and explanations of patients’ conditions within limited time (14), they constitute a high-risk group for burnout. Previous research has shown that burnout not only reduces individual job satisfaction, psychological resilience, and occupational persistence (15, 16) but is also closely associated with medical errors, deterioration of service attitudes, increased turnover intention, and impaired teamwork (17, 18). For healthcare institutions, the long-term accumulation of burnout among healthcare workers may lead to the loss of human resources, declining organizational performance, and reduced operational efficiency of the healthcare system (19, 20). Effective identification of the factors influencing burnout is therefore particularly important. Recent studies suggest that workplace violence may be regarded as a potent occupational stressor that not only directly elicits negative emotional reactions such as fear, anger, humiliation, and helplessness (21, 22), but may also disrupt healthcare workers’ fundamental perceptions of the controllability and fairness of their work environment. According to the Job Demands–Resources model (23), exposure to violence markedly increases emotional job demands and depletes individual psychological resources (24); when organizational support, coworker support, and safety safeguards are insufficient, resource compensation mechanisms fail to function, ultimately aggravating burnout. At the same time, burnout may diminish healthcare workers’ capacity for emotional regulation, communication tolerance, and conflict coping, making them more prone to becoming embroiled in tense situations during complex doctor–patient interactions and thereby further increasing the risk of the occurrence or perception of workplace violence. The relationship between workplace violence and burnout may therefore not be a simple unidirectional causal one, but rather a complex association characterized by mutual reinforcement and co-clustering.

Most previous studies have examined the prevalence, correlates, and health consequences of workplace violence or burnout from a variable-centered perspective (25, 26). Such research treats the level of workplace violence or burnout as a continuous variable or a single outcome, providing important evidence for understanding overall associations. However, the healthcare workforce is markedly heterogeneous, with different individuals potentially exhibiting concurrent violence exposure and burnout of varying types and intensities; comparing average levels alone may obscure latent high-risk subgroups. Moreover, both workplace violence and burnout possess multidimensional structures, and analyzing any single dimension in isolation cannot adequately capture their joint distributional characteristics. To address these limitations, the present study employs latent profile analysis to uncover patterns of coexistence between workplace violence and burnout. For instance, some healthcare workers may simultaneously exhibit low violence exposure and low burnout, whereas others may experience high violence exposure without a corresponding marked elevation in burnout. Identifying these differentiated profiles facilitates a shift from “average risk” to “precise identification, “ providing a basis for prioritizing subsequent interventions and allocating resources.

Among the factors influencing these joint latent profiles, work–family conflict and emotional labor represent important psychosocial determinants. Work–family conflict refers to a state of incompatibility between work roles and family roles in terms of time, energy, and behavioral demands, and is common in healthcare environments characterized by frequent shift work, night shifts, overtime, emergency resuscitations, and holiday duty (27, 28). Prolonged work–family conflict depletes individual recovery resources, weakens the buffering effect of family support (29, 30), and increases the accumulation of negative emotions, rendering healthcare workers more susceptible to emotional exhaustion and occupational disengagement. Furthermore, when individuals are unable to recover adequately because of family responsibilities, or experience guilt and stress because work intrudes upon family life, their patience, empathy, and communication efficacy in clinical interactions may decline, thereby increasing the likelihood of doctor–patient conflict. Emotional labor, in turn, refers to the process by which healthcare workers regulate, suppress, or feign their own emotions in accordance with occupational role requirements in order to maintain a professional image and adhere to service norms (31, 32). Surface acting often requires individuals to mask their genuine feelings, and its sustained use may lead to emotional dysregulation and psychological exhaustion (33, 34); deep acting, although relatively more adaptive, may still impose resource depletion in high-pressure situations (35). When healthcare workers must remain calm, empathetic, and professional even while encountering verbal abuse, questioning, or even threats, the burden of emotional labor markedly amplifies the effect of violence experiences on burnout. Work–family conflict and emotional labor may therefore serve not only as direct predictors of burnout but also as factors shaping different joint profiles of violence and burnout.

In addition to work-related psychosocial factors, demographic and occupational characteristics may also influence the probability of healthcare workers being assigned to different risk profiles. Sex differences may manifest in exposure to types of violence, norms of emotional expression, family role expectations, and coping strategies (36, 37). Specifically, female healthcare workers may shoulder greater family caregiving responsibilities and face higher emotional labor demands (38), whereas male healthcare workers may bear different risks in physical conflicts or threatening situations (39). Age and years of service represent both the accumulation of occupational experience, communication skills, and emergency-handling capacity, and may also reflect resource depletion resulting from long-term exposure to occupational stress (40). Educational level may influence job responsibilities, occupational expectations, problem-solving capacity, and organizational status, and is thus associated with the perception of violence and the level of burnout. Physicians and nurses differ in work content, frequency of patient contact, decision-making responsibility, and role boundaries. Nurses typically spend more time in contact with patients and families and may be more frequently exposed to verbal aggression and service-related conflicts, whereas physicians may bear greater responsibility-related stress arising from diagnostic and treatment decisions, therapeutic outcomes, and explanations of costs. More importantly, health-related behaviors such as smoking and alcohol consumption may serve both as means of coping with stress and as overt markers of accumulated psychological load. Children’s status is closely related to family responsibilities, time allocation, and recovery opportunities; particularly against the backdrop of high-intensity medical work, parenting stress may further exacerbate work–family conflict. Incorporating these factors into the analysis therefore contributes to a more comprehensive explanation of the mechanisms underlying the formation of different joint violence–burnout profiles.

The present study targets healthcare workers and adopts a cross-sectional design to conduct a joint profile analysis of workplace violence and burnout, and further examines the influence of work–family conflict, emotional labor, and demographic and occupational factors on membership in different latent profiles. The study seeks to achieve the following objectives:

1. to identify, from a person-centered perspective, the co-occurrence patterns of workplace violence and burnout among healthcare workers, thereby addressing the inability of prior variable-centered research to reveal population heterogeneity;
2. to comprehensively incorporate key psychosocial variables such as work–family conflict and emotional labor, thereby deepening understanding of the mechanisms linking violence exposure and psychological exhaustion;
3. to integrate factors including sex, age, educational level, years of service, occupational role, smoking, alcohol consumption, and children’s status into a single analytical framework, in order to characterize different risk subgroups and provide a basis for precision intervention.

Through these aims, this study is intended to provide evidentiary support for healthcare institutions in establishing stratified early-warning systems, optimizing organizational support strategies, improving the doctor–patient communication environment, and promoting the mental health of healthcare workers.

## 2 Methods

### 2.1 Study design

This study employed a multicenter cross-sectional survey design, recruiting healthcare workers from six hospitals in Sichuan Province, China, between April and May 2026, with the aim of conducting a joint profile analysis of workplace violence and burnout and identifying their influencing factors. The study strictly adhered to the ethical principles of the Declaration of Helsinki and was reviewed and approved by the Medical Ethics Committee of the Affiliated Hospital of North Sichuan Medical College. All participants read and signed an electronic informed consent form before formally completing the questionnaire.

### 2.2 Study participants

This study used convenience sampling, with the target population being on-duty healthcare workers directly engaged in clinical medical and nursing work in general hospitals. Using clinical departments as sampling units within the participating institutions, the researchers covered major clinical departments including internal medicine, surgery, emergency medicine, and critical care medicine. The researchers explained the study’s purpose, content, and risks in detail to the department heads and, upon obtaining their consent, distributed the electronic questionnaire. The QR code for the electronic questionnaire was generated by a professional data-collection platform.

To enhance the rigor of the study, the inclusion criteria were as follows: (1) holding a valid physician’s or nurse’s practicing certificate; (2) having been continuously engaged in clinical work at the current institution for ≥ 8 months; (3) being directly involved in patient diagnosis, treatment, or nursing care, with routine doctor–patient/nurse–patient contact; (4) aged ≥18 years; and (5) providing informed consent to participate and being able to independently complete the online self-report questionnaire.

The exclusion criteria were: (1) being off-duty for ≥1 month during the survey period because of sick leave, maternity leave, personal leave, or external training; (2) hospital staff working in non–direct patient-contact positions such as administrative management, logistics support, or pharmacy; (3) intern physicians or intern nurses in the standardized training stage who had not yet obtained independent practicing qualifications; (4) inability to complete the questionnaire independently because of cognitive impairment, mental illness, severe physical illness, or other reasons; and (5) excessively short questionnaire completion time or an evident regular (patterned) response style.

#### 2.2.1 Minimum sample size estimation

The sample size required for latent profile analysis is influenced by factors such as the number of profile indicators, the number of latent classes, the degree of class separation, and the strength of the associations between indicators and classes. Drawing on the simulation studies of Nylund-Gibson and Choi (2018) and Tein, Coxe (41) regarding sample size requirements for latent profile analysis, when the number of profile indicators is 5–10, the number of latent classes is 2–4, and classification quality is ≥0.80, an effective sample size of ≥300–500 ensures model convergence stability and accuracy of parameter estimation. According to the empirical rule for multivariable logistic regression sample size proposed by Peduzzi, Concato (42)—namely, that the number of events per predictor variable should be no fewer than 10—and given that this study anticipated including 10–15 predictor variables in the logistic regression model, the most conservative calculation based on 15 predictors yields a minimum required number of events of 150. Assuming that the high-risk profile accounts for 50% of the sample, the minimum total sample size required is therefore 300.

A total of 643 healthcare workers were approached in this study. Of these, 14 were on sick leave, personal leave, or external training; 8 had not yet completed their internship as intern physicians or intern nurses; and 5 participants were identified during data cleaning as having provided patterned responses. Ultimately, 616 healthcare workers were included as the valid analytic sample, yielding an effective response rate of 95.8%.

### 2.3 Measure tools

#### 2.3.1 Workplace violence scale

The items measuring workplace violence were derived from the Chinese version of the Workplace Violence Scale adapted by Wang, Wang (43). This scale comprises five items and is unidimensional, covering physical assault, emotional abuse, threats, verbal sexual harassment, and sexual abuse. A sample item is: “Do you agree that you have experienced physical assault (e.g., spitting, biting, hitting, pushing) during work?” The scale has been widely used among Chinese healthcare workers (44). In the present study, it was used to measure workplace violence among Chinese healthcare workers, with responses scored on a 5-point Likert scale ranging from “1 = strongly disagree” to “5 = strongly agree.” Total scores range from 5 to 25, with higher scores indicating a more severe perception of workplace violence experienced by healthcare workers. In this study, the Cronbach’s α coefficient of the scale was 0.874, demonstrating good internal consistency and reliability.

#### 2.3.2 Burnout scale

The items measuring burnout were derived from the Chinese version of the Oldenburg Burnout Inventory adapted by Xu, Yuan (45). This scale is divided into two dimensions—exhaustion (8 items) and disengagement (8 items)—comprising 16 items in total. The scale was initially used to assess Chinese nurses in order to verify its cultural applicability and reliability, and it has been further validated in studies such as those by Zhang, Xiao (46) and Huang, Huang (47). The present study used this scale to assess burnout among Chinese healthcare workers; a sample item is: “Do you agree that there are days when you feel tired before you arrive at work?” Responses were scored on a 5-point Likert scale (1 = strongly disagree, 5 = strongly agree), with total scores ranging from 16 to 80. Higher scores indicate stronger feelings of burnout among healthcare workers. In this study, the Cronbach’s α coefficient of the scale was 0.732, demonstrating good internal consistency and reliability.

#### 2.3.3 Work–family conflict scale

The items measuring work–family conflict were derived from the multidimensional Work–Family Conflict Scale developed by Carlson, Kacmar (48). This scale comprises 18 items across six dimensions: time-based work interference with family, time-based family interference with work, strain-based work interference with family, strain-based family interference with work, behavior-based work interference with family, and behavior-based family interference with work. A sample item is: “Do you agree that the time you spend on family responsibilities often interferes with your involvement in work activities that would benefit your career?” The scale was adapted into Chinese by Jung and Kim (49), and its cultural adaptability and reliability were validated in a Chinese population. The present study used this scale to measure work–family conflict among healthcare workers, with responses scored on a 5-point Likert scale (“1 = strongly disagree, “ “5 = strongly agree”). Total scores range from 18 to 90, with higher scores indicating more severe work–family conflict. In this study, the Cronbach’s α coefficient of the scale was 0.781, demonstrating good internal consistency.

#### 2.3.4 Emotional labor scale

The items measuring emotional labor were derived from the Chinese version of the Emotional Labor Scale developed by Yang, Chen (50). This scale comprises 12 items, covering surface acting, deep acting, natural emotional expression, and emotional suppression. A sample item is: “Do you agree that, when patients make excessive demands or when you are temporarily unable to meet their needs during work, you feel helpless?” The scale has been widely applied among Chinese healthcare workers; for example, Huang, Zheng (51) used it to measure emotional labor among psychiatric nurses. The present study used this scale to measure emotional labor among Chinese healthcare workers, with responses scored on a 5-point Likert scale ranging from “1 = strongly disagree” to “5 = strongly agree.” Total scores range from 12 to 60, with higher scores indicating a greater tendency for healthcare workers to adopt emotional labor strategies. In this study, the Cronbach’s α coefficient of the scale was 0.926, demonstrating good internal consistency.

### 2.4 Statistical analysis

Data were analyzed using SPSS 27.0 and Mplus 8.3, with the significance level set at 0.05. First, we conducted routine analyses including reliability testing, assessment of common method bias, descriptive statistics, and correlation analysis.

Class enumeration was performed on 21 continuous indicators: the five items of the Workplace Violence Scale and the 16 items of the Chinese version of the Oldenburg Burnout Inventory. Item-level indicators were retained in the primary model for three reasons. First, the Workplace Violence Scale is a behaviourally heterogeneous exposure checklist rather than a homogeneous attitudinal scale: the five acts differ substantially in base rate and severity, and collapsing them into a single parcel would obscure precisely the type-specific exposure configurations that a person-centred analysis is intended to recover. Second, parcelling all 21 items into three dimension scores leaves only three indicators, which constrains the model’s capacity to detect shape (qualitative) as opposed to purely level (quantitative) differences between profiles and reduces the information available for class separation. Third, the primary model remained well identified and empirically stable.

Class enumeration criteria. Models with one to five classes were estimated with robust maximum likelihood (MLR), 5, 000 random start values and 250 final-stage optimisations (STARTS = 5000 250; maximum 500 iterations). The optimal number of profiles was determined by jointly considering five pre-specified criteria rather than any single index: (a) information criteria (AIC, BIC, aBIC), evaluated by the magnitude of successive decrements (“elbow” criterion) rather than by the absolute minimum, because information criteria frequently decline monotonically in empirical mixture applications; (b) likelihood-ratio tests, interpreting the LMR adjusted LRT and the BLRT in the light of their known behaviour—the BLRT has greater power but is prone to over-extraction when continuous indicators depart from within-class normality or when local independence is imperfect; (c) classification quality (entropy ≥ 0.80; average posterior probability ≥ 0.90 for each class); (d) minimum class size, with any class comprising < 5% of the sample or fewer than 25 cases treated as potentially spurious and not retained; and (e) substantive interpretability and parsimony, i.e., whether an additional class represented a qualitatively distinct configuration of violence and burnout rather than a further gradation of severity.

After determining the optimal latent profile solution, we compared the differences among the profiles with respect to demographic characteristics, occupational characteristics, health-related behaviors, and psychosocial factors, in order to preliminarily characterize the external features of each profile. For categorical variables, the χ² test was used to compare distributional differences among the profiles; for continuous variables, the independent-samples t-test was used. To further identify the independent predictors influencing healthcare workers’ assignment to different latent profiles, a binary logistic regression model was constructed with the class membership extracted from the optimal latent profile solution as the dependent variable. Variables that showed a significant effect in the univariate analyses were entered into the binary logistic regression model. Categorical independent variables were coded as dummy variables in the regression model, with a clearly defined reference group specified for each variable. Because the “technical secondary school/high school” category of educational level contained only two participants, which can induce quasi-complete separation and unstable maximum-likelihood estimates in logistic regression, this category was merged with the adjacent “junior college” category to form a combined “junior college or below” reference category. As a sensitivity analysis, Firth’s penalized (bias-reduced) logistic regression (logistf package, R 4.6.0) was also fitted to the original four-category educational variable to confirm that the recoding did not alter the substantive conclusions. For the continuous work–family conflict score, we additionally report the odds ratio per one-standard-deviation (SD) and per interquartile-range (IQR) increase to facilitate interpretation of the practical effect size.

## 3 Results

### 3.1 Demographic characteristics

A total of 616 healthcare workers were included in this study, comprising 421 nurses (68.3%) and 195 physicians (31.7%). There were 83 male participants (13.5%) and 533 female participants (86.5%), with a mean age of 36.80 ± 7.547 years, indicating that the sample consisted predominantly of mid-career healthcare workers. As shown in **Table 1**, the sample was predominantly composed of healthcare workers who were female, nurses, holders of a bachelor’s degree or higher, married, with children, employed under contract/appointment arrangements, and working in tertiary Grade-A hospitals.

**Table 1:** Demographic characteristics of the study sample.

Variables	Items	Frequency (N)	Proportion (%)
Hospital Role	The doctor	195	31.7%
	Nurse	421	68.3%
Gender	Male	83	13.5%
	female	533	86.5%
Educational background	Technical secondary school/high school	2	0.3%
	Junior college	55	8.9%
	Undergraduate college	397	64.4%
	Master degree or above	162	26.3%
Marital status	Unmarried	93	15.1%
	Married	506	82.1%
	Get divorced	17	2.8%
Status of children	Have children	461	74.8%
	No children at present	155	25.2%
Nature of employment	Establishment of institutions	160	26.0%
	Contract/appointment system	434	70.5%
	Dispatch of labor services	22	3.6%
Grade of hospital	Grade 3 A	614	99.7%
	Grade 3 B/Grade 2 A	1	0.2%
	Secondary Class B and below/community health service centers	1	0.2%
Monthly income level	6, 000 yuan or less	203	33.0%
	6001–9000 yuan	280	45.5%
	¥9001 and above	133	21.6%
Length of service	8 months to 1year	12	1.9%
	1 to 3 years	22	3.6%
	3 to 5 years	53	8.6%
	5 years or more	529	85.9%
Smoking	Yes.	23	3.7%
	No.	593	96.3%
Drinking alcohol	Yes	120	19.5%
	No	496	80.5%
Age	Year	36.80 ± 7.547	

### 3.2 Common method bias

This study employed a cross-sectional questionnaire design, and the core variables were all derived from participants’ self-reports, which may give rise to common method bias. To assess the potential influence of common method bias, Harman’s single-factor test was used to conduct an unrotated exploratory factor analysis on the measurement items of all core variables. The results revealed nine factors with eigenvalues greater than 1, and the first factor accounted for 28.508% of the variance, which is below the 40% threshold.

During data collection, the researchers emphasized the anonymity, confidentiality, and security of the data in order to further reduce common method bias. Nevertheless, cross-sectional self-report data may still be subject to social desirability, recall bias, and common-source measurement error, so a degree of caution should still be exercised when interpreting the relationships among the variables.

### 3.3 Descriptive statistics and correlation analysis

The descriptive statistics for the core study variables are presented in **Table 2**. The means of the core variables ranged from 2.000 to 3.432. Among these, healthcare workers’ scores for emotional labor and burnout were both higher than the theoretical midpoint of their respective scales, suggesting that emotional-regulation load and occupational psychological exhaustion may be fairly prevalent in high-intensity medical service environments.

**Table 2:** Descriptive statistics of the core study variables.

Variables	M	SD	Skewness	Kurtosis
Workplace violence	2.000	1.045	0.651	-0.797
Burnout	3.195	0.437	-0.907	6.175
Emotional labor	3.432	0.454	-0.577	5.100
Work-family conflict	2.885	0.700	-0.238	1.052

Skewness ranged from -0.907 to 0.651 and kurtosis from -0.797 to 6.175. Although the absolute values of skewness lie within conventionally acceptable limits (52), burnout (kurtosis = 6.175) and emotional labour (kurtosis = 5.100) were clearly leptokurtic: responses to these scales were tightly concentrated around a central peak (burnout: M = 3.195, SD = 0.437) with a limited number of extreme values in the tails. Floor and ceiling responses were nevertheless uncommon (0.6% of participants at the scale minimum and 0.2% at the scale maximum for burnout), suggesting that the leptokurtosis reflects central-tendency responding rather than a strict ceiling or floor effect. Because latent profile analysis with continuous indicators assumes within-class normality, this departure from normality is not fully addressed by MLR estimation, robust estimation corrects standard errors and test statistics but does not protect class enumeration, and non-normal indicators are known to promote the extraction of spurious classes (53). This consideration was explicitly incorporated into our class enumeration strategy and provides a further, distributional explanation for the persistent significance of the BLRT beyond two classes; it therefore reinforces, rather than undermines, the decision to retain the parsimonious two-class solution.

Subsequently, we conducted a correlation analysis between the core study variables and the demographic information, as shown in **Table 3** and **Figure 1**. The results indicated that workplace violence was significantly and positively correlated with burnout (r = 0.334, p < 0.001); workplace violence was not significantly correlated with emotional labor (r = 0.068, p = 0.091); and workplace violence was significantly correlated with work-family conflict (r = 0.285, p < 0.001). Burnout was significantly and positively correlated with emotional labor (r = 0.471, p < 0.001), and significantly and positively correlated with work-family conflict (r = 0.468, p < 0.001). Emotional labor was not significantly correlated with work-family conflict (r = 0.195, p < 0.001).

**Table 3:** Correlation analysis among the core study variables and demographic information.

Variables	1	2	3	4	5	6	7	8	9	10	11	12	13	14	15	16
1. Workplace violence	1															
2. Burnout	0.334**	1														
3. Emotional labor	0.068	0.471**														
4. work-family conflict	0.285**	0.468**	0.195**													
5. Hospital Role	-0.199**	-0.093*	0.061	-0.161**												
6. Gender	-0.192**	-0.045	0.051	-0.157**	0.559**											
7. Age	0.124**	0.108**	0.163**	0.075	-0.306**	-0.166**										
8. Educational background	0.196**	0.126**	0.014	0.198**	-0.717**	-0.410**	0.248**									
9. Marital status	-0.022	0.03	0.114**	0.082*	0.008	-0.015	0.376**	0.06								
10. Status of children	-0.027	-0.043	-0.147**	-0.051	0.025	0.032	-0.472**	-0.057	-0.647**							
11. Nature of employment	-0.105**	-0.118**	-0.102*	-0.135**	0.397**	0.167**	-0.590**	-0.429**	-0.252**	0.270**						
12. Grade of hospital	0.055	0.025	-0.078	0.02	-0.080*	0.021	-0.015	0.078	0.017	0.01	-0.012					
13. Monthly income level	0.106**	0.137**	0.008	0.112**	-0.575**	-0.322**	0.391**	0.504**	0.090*	-0.125**	-0.421**	-0.041				
14. Length of service	0.05	0.126**	0.148**	0.128**	0.151**	0.064	0.400**	-0.027	0.394**	-0.441**	-0.306**	-0.041	0.044			
15. Smoking	-0.051	0.035	0.049	-0.04	0.105**	0.273**	-0.067	-0.061	-0.039	0.035	-0.02	0.011	-0.031	0.000		
16. Drinking alcohol	-0.051	-0.019	-0.038	-0.085*	0.044	0.250**	-0.043	-0.105**	-0.049	0.077	0.018	0.027	-0.088*	-0.02	0.271**	

*p < 0.05; **p<0.01.

**Figure 1:**
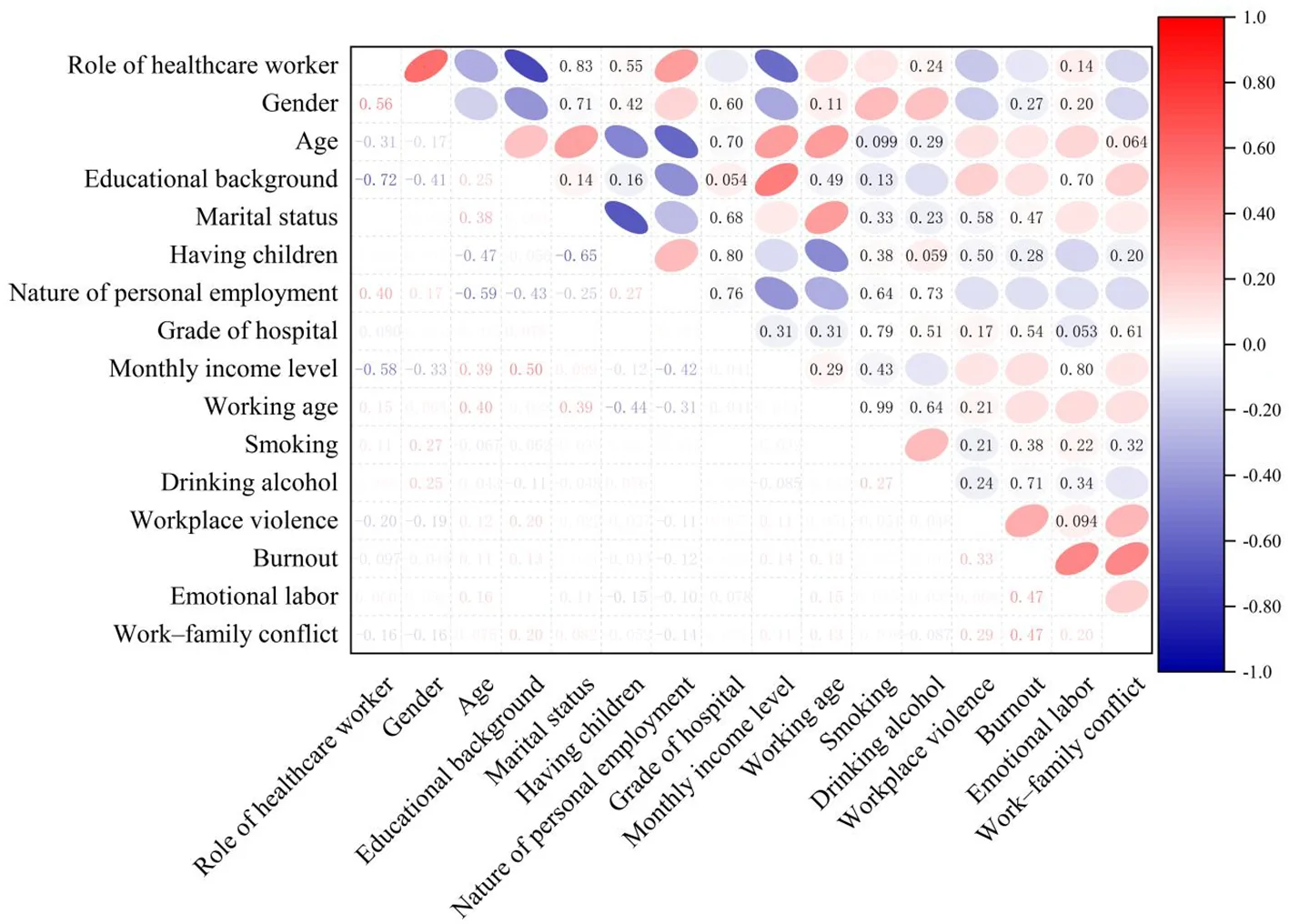
Correlation analysis among the core study variables and demographic information. The figures in the upper-right corner represent significance levels; the figures in the lower-left corner represent correlation coefficients.

### 3.4 Joint latent profile analysis of workplace violence and burnout

To identify the joint distribution patterns of workplace violence and burnout among healthcare workers, this study used the indicators related to workplace violence and burnout as profile indicators and fitted latent profile models with one to five classes in sequence. The optimal model was determined by comprehensively comparing the AIC, BIC, aBIC, entropy, the LMR test, the BLRT test, and the sample proportion of each class, as shown in **Table 4**.

**Table 4:** Fit indices for the 1- to 5-class latent profile models (Measure items).

Index	Profile 1	Profile 2	Profile 3	Profile 4	Profile 5
Free parameters	42	64	86	108	130
Log-likelihood	-19797.752	-18376.095	-17838.555	-17370.726	-17024.198
AIC	39679.504	36880.189	35849.111	34957.453	34308.396
BIC	39865.280	37163.277	36229.510	35435.164	34883.418
ΔBIC	-	2702.003	933.767	794.346	551.746
aBIC	39731.938	36960.089	35956.476	35092.284	34470.693
Entropy	-	0.907	0.912	0.926	0.940
LMR (p)	-	< 0.001	0.156	0.214	0.108
BLRT (p)	-	< 0.001	< 0.001	< 0.001	< 0.001
Class 1	-	47.08%	17.70%	26.46%	15.58%
Class 2	-	52.92%	53.08%	25.65%	11.53%
Class 3	-	-	29.22%	30.68%	31.49%
Class 4	-	-	-	17.21%	25.16%
Class 5	-	-	-	-	16.23%

As shown in **Table 4**, AIC, BIC and aBIC decreased monotonically from the one- to the five-class solution. The entropy values for the 2- through 5-class models were all above 0.80, indicating that all of these models had good classification accuracy. However, the decrement was by far the largest at the transition from one to two classes (ΔBIC = 2702.003) and diminished progressively thereafter (ΔBIC = 933.767, 794.346 and 551.746 for the three-, four- and five-class solutions, respectively), yielding a distinct elbow at two classes. However, in terms of the likelihood ratio tests, the LMR test reached statistical significance only for the 2-class model (p < 0.001), whereas the LMR tests for the 3-, 4-, and 5-class models did not reach significance. This indicates that, compared with the 1-class model, the 2-class model significantly improved model fit, but adding further classes beyond the 2-class model was not supported by the LMR test. The BLRT test reached statistical significance for the 2- through 5-class models (all p < 0.001), suggesting that adding classes might still yield statistical improvements in fit.

The average posterior classification probabilities further showed that the average probability of individuals in Class 1 being correctly assigned to Class 1 was 0.972, and the average probability of individuals in Class 2 being correctly assigned to Class 2 was 0.972. The average posterior probabilities for both classes exceeded 0.90, indicating that the extracted latent profiles had high classification reliability and a low risk of misclassification, as shown in **Table 5**.

**Table 5:** Average latent class probabilities for most likely latent class membership.

Class	Class 1	Class 2
Class 1	0.972	0.028
Class 2	0.028	0.972

In the 2-class model, Class 1 comprised 290 healthcare workers (47.08%), and Class 2 comprised 326 healthcare workers (52.92%), as shown in **Figure 2**. Based on the subsequent results regarding between-profile variable differences and the principal profile characteristics, Class 1 was primarily characterized by a lower level of work-family conflict together with a slightly higher level of emotional labor, and was therefore labeled the “relatively adaptive profile.” Class 2, by contrast, exhibited a markedly higher level of work-family conflict, and the subsequent regression analysis showed that its membership probability was strongly driven by elevated work-family conflict; it was therefore labeled the “high-risk profile.”

**Figure 2:**
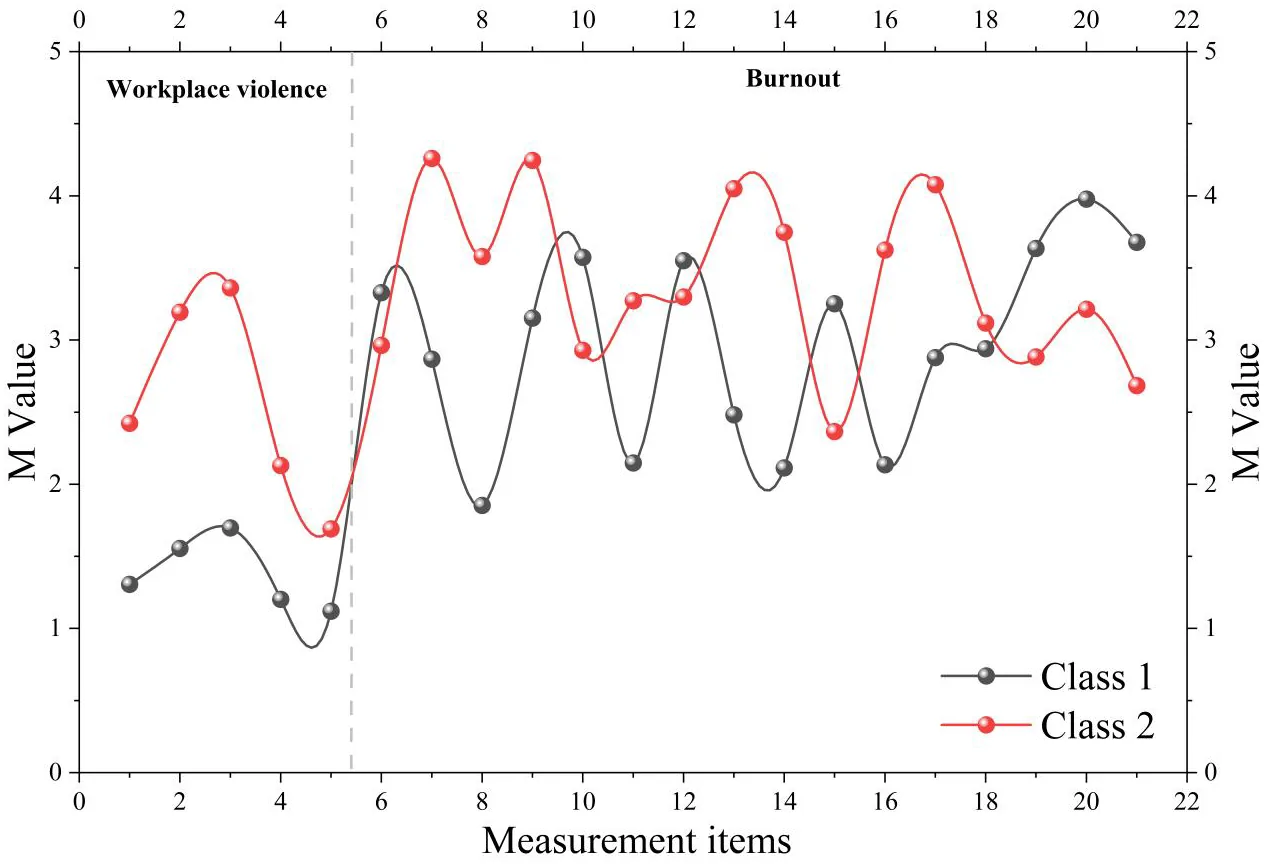
Profile of the optimal latent profile of workplace violence and burnout. 1 to 5 measurement items entitled workplace violence; 6–21 measurement items entitled burnout.

Parcel-level sensitivity analysis. When the same model sequence was re-estimated with three parcel indicators, the two-class solution was again supported (AIC = 3301.036, BIC = 3345.268, aBIC = 3313.520, entropy = 0.872; LMR p < 0.001), whereas the 3-, 4- and 5-class solutions each contained a class comprising only 1.46% of the sample (n = 9), below the conventional minimum-class-size threshold of 5% (or 25 cases) and therefore regarded as substantively uninterpretable and potentially spurious (**Table 6**).

**Table 6:** Fit indices for the 1- to 5-class latent profile models (Measurement dimension).

Index	Profile 1	Profile 2	Profile 3	Profile 4	Profile 5
AIC	3590.145	3301.036	3082.472	2970.633	2893.329
BIC	3616.685	3345.268	3144.398	3050.251	2990.641
aBIC	3597.636	3313.520	3099.950	2993.105	2920.795
Entropy	-	0.872	0.919	0.905	0.909
LMR (p)	-	< 0.001	< 0.001	0.037	0.096
BLRT (p)	-	< 0.001	< 0.001	< 0.001	< 0.001
Class 1	-	62.34%	1.46%	28.73%	54.22%
Class 2	-	37.66%	37.34%	1.46%	29.55%
Class 3	-	-	61.20%	55.68%	1.46%
Class 4	-	-	-	14.12%	12.99%
Class 5	-	-	-	-	1.79%

The parcel-level solution did, however, yield a smaller high-risk class (37.66%) than the item-level solution (52.92%). This indicates that, although the number, ordering and qualitative configuration of the joint profiles are robust to indicator specification, the estimated prevalence of the high-risk profile is not, and prevalence figures should therefore be interpreted as an approximate range (roughly 38–53%) rather than a point estimate.

### 3.5 Univariate analysis of demographic information across the optimal latent profiles

To further characterize the different latent profiles, this study compared Class 1 and Class 2 with respect to demographic variables, occupational variables, health-related behaviors, and psychosocial factors. The results showed that the two groups of healthcare workers differed significantly in terms of hospital role (χ² = 24.984, p < 0.001), gender (χ² = 14.441, p < 0.001), educational level (χ² = 22.013, p < 0.001), monthly income level (χ² = 13.412, p = 0.001), and smoking (χ² = 4.225, p = 0.040), whereas the differences in marital status, parental status, type of employment, hospital grade, years of work experience, drinking, and age were not statistically significant (p > 0.05), as shown in **Table 7**.

**Table 7:** Univariate analysis of demographic information on latent profiles.

Variables	Items	Class1	Class 2	χ²/t	p
Hospital Role	Doctor	63	132	24.984	<0.001
	Nurse	227	194		
Gender	Male	23	60	14.441	<0.001
	female	267	266		
Educational background	Technical secondary school/high school	1	1	22.013	<0.001
	Junior college	34	21		
	Undergraduate college	203	194		
	Master degree or above	52	110		
Marital status	Unmarried	38	55	1.859	0.395
	Married	243	263		
	Get divorced	9	8		
Status of children	Have children	219	242	0.134	0.714
	No children at present	71	84		
Nature of employment	Establishment of institutions	65	95	3.864	0.145
	Contract/appointment system	213	221		
	Dispatch of labor services	12	10		
Grade of hospital	Grade 3 A	290	324	1.785	0.410
	Grade 3 B/Grade 2 A	0	1		
	Secondary Class B and below/community health service centers	0	1		
Monthly income level	6, 000¥ or less	113	90	13.412	0.001
	6001-9000¥	130	150		
	¥9001 and above	47	86		
Length of service	8 months to 1year	9	3	4.168	0.244
	1 to 3 years	9	13		
	3 to 5 years	24	29		
	5 years or more	248	281		
Smoking	Yes.	6	17	4.225	0.040
	No.	284	309		
Drinking alcohol	Yes	51	69	1.254	0.263
	No.	239	257		
Age	Year.	36.82 ± 7.736	36.79 ± 7.387	0.053	0.958

The comparison of psychosocial factors showed that the emotional labor score was 3.399 ± 0.501 for Class 1 and 3.462 ± 0.407 for Class 2, with a statistically not significant difference between the two groups (t = 1.724, p = 0.085). By contrast, work-family conflict showed a far more pronounced difference between the two classes: the score was 2.501 ± 0.677 for Class 1 and 3.227 ± 0.521 for Class 2, with a highly statistically significant difference (t = 15.000, p < 0.001). Therefore, work-family conflict may be the core psychosocial factor distinguishing the two latent profiles. Class 2 healthcare workers had a significantly elevated level of work-family conflict, indicating that they were more likely to be in a state in which work and family roles mutually encroach on one another, recovery resources are insufficient, and role-related stress accumulates, as shown in **Table 8** and **Figure 3**.

**Table 8:** Univariate analysis of demographic information across the latent profiles.

Variables	Class1 (M ± SD)	Class 2 (M ± SD)	t	p	Cohens’s d
Emotional labor	3.399 ± 0.501	3.462 ± 0.407	1.724	0.085	0.139
Work-family conflict	2.501 ± 0.677	3.227 ± 0.521	15.000	<0.001	1.211

**Figure 3:**
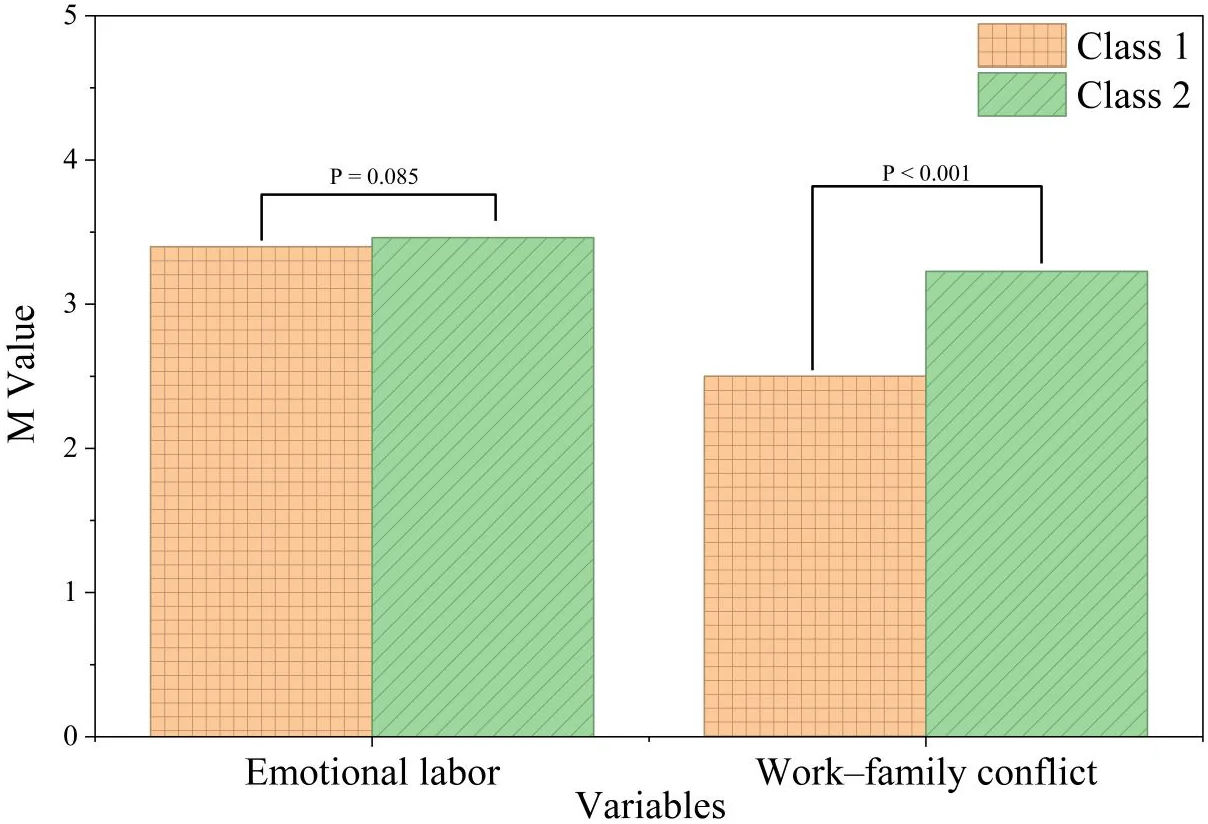
Differences in the optimal latent profile for emotional labor and work-family conflict.

### 3.6 Binary logistic regression analysis

To further identify the independent factors influencing healthcare workers’ assignment to different latent profiles, this study conducted a binary logistic regression analysis with latent profile membership as the dependent variable, as shown in **Table 9**. Based on the results of the preceding univariate analyses, hospital role, gender, educational level, monthly income level, smoking, and work-family conflict were entered into the model. Educational level was recoded as a three-category variable (“junior college or below”, “undergraduate college”, and “master degree or above”) to avoid quasi-complete separation caused by the near-empty “technical secondary school/high school” category (n = 2). With Class 1 as the reference category, the effects of each factor on assignment to Class 2 were examined.

**Table 9:** Analysis of the independent influencing factors of the different latent profiles.

Variables	Items	B	SE	P	OR	95% CI
Hospital Role	Nurse	−0.584	0.405	0.149	0.558	0.249–1.223
	Doctor (reference)					
Gender	Female	−0.084	0.359	0.815	0.919	0.450–1.851
	Male (reference)					
Educational background	Undergraduate college	0.036	0.354	0.918	1.037	0.518–2.086
	Master degree or above	−0.037	0.514	0.942	0.963	0.349–2.637
	Junior college or below (reference)					
Monthly income level	6001–9000 CNY	0.129	0.232	0.578	1.137	0.722–1.792
	≥9001 CNY	0.245	0.342	0.474	1.278	0.652–2.498
	≤6000 CNY (reference)					
Smoking	No	−0.975	0.603	0.106	0.377	0.107–1.164
	Yes (reference)					
Work–family conflict	(per 1-unit increase)	2.365	0.229	<0.001	10.647	6.940–17.029

Work–family conflict was entered as a continuous score (mean of 18 items, 1–5). Because a one-unit change on the 5-point scale is large, the odds ratio scaled to the observed dispersion is also reported: OR per one-SD increase (SD = 0.70) = 5.24 (95% CI 3.83–7.17); OR per one-IQR increase (IQR = 0.72) = 5.52 (95% CI 3.99–7.63). Educational background was recoded by merging the “technical secondary school/high school” category (n = 2) with “junior college” to form the reference category “junior college or below”, thereby resolving quasi-complete separation; a Firth penalized regression sensitivity analysis on the original four-category variable yielded materially identical conclusions (all p > 0.79 for education).

Work-family conflict was the strongest independent predictor of assignment to Class 2. For each one-unit increase in work-family conflict (a 5-point Likert mean), the likelihood of a healthcare worker being assigned to Class 2 increased significantly (B = 2.365, p < 0.001, OR = 10.647, 95% CI: 6.940–17.029). Because a one-unit change spans a large portion of the 5-point scale, we additionally report the effect scaled to the observed dispersion of the score: each one-standard-deviation increase in work-family conflict (SD = 0.70) was associated with a 5.24-fold increase in the odds of Class 2 membership (95% CI: 3.83–7.17), and each interquartile-range increase (IQR = 0.72) with a 5.52-fold increase (95% CI: 3.99–7.63). This result indicates that, with other variables held constant, the higher the level of work-family conflict, the more likely a healthcare worker was to belong to the high-risk latent profile. None of the other variables reached statistical significance in the multivariable model (p > 0.05).

Therefore, work-family conflict was the core independent risk factor for healthcare workers’ assignment to Class 2. By comparison, demographic and general occupational characteristics had limited independent predictive value in the multivariable model.

## 4 Discussion

### 4.1 Joint latent profile analysis of workplace violence and burnout

Person-centred approaches have previously been applied to workplace violence and to burnout separately, for example, latent profile analyses of violence exposure among nurses (54) and of burnout subtypes among Chinese nursing populations (55). To our knowledge, however, the present study is among the first to model workplace violence and burnout jointly within a single latent profile solution among healthcare workers, and thereby to characterise their co-occurrence. By systematically comparing the fit indices of the 1- to 5-class latent profile models, this study ultimately selected the 2-class model as the optimal solution.

In the optimal 2-class model, Class 1 was labeled the “relatively adaptive profile.” This subgroup scored at relatively low levels on all dimensions of workplace violence and burnout, suggesting that this group may possess relatively ample reserves of adaptive psychological resources, enabling them to maintain a relatively healthy state of psychological functioning when confronting the emotional challenges of clinical work. By contrast, Class 2 was labeled the “high-risk profile.” This subgroup showed significantly elevated scores across all dimensions of violence and burnout, accompanied by a prominent level of work-family conflict, indicating a marked clustering of overall mental health risk. More than half of the healthcare workers belonged to the high-risk profile, a proportion far higher than the detection rates of high-risk burnout populations reported in previous single-dimension studies. This difference may stem from the fact that joint profile analysis can capture the cumulative and synergistic features of occupational mental health risk in a more comprehensive and sensitive manner. When workplace violence and burnout act synergistically on the same individual, their additive effect may push more healthcare workers across the clinical risk threshold.

According to the Job Demands–Resources model, workplace violence continuously depletes an individual’s psychological resources (24); when resource depletion exceeds an individual’s capacity for compensation and recovery, burnout develops via a health-impairment pathway (56). Conservation of Resources theory further points out that individuals not only strive to protect their existing resources but also actively invest resources to cope with the threat of future resource loss (57). Therefore, Class 1 healthcare workers may possess more ample personal resources (such as resilience and emotion-regulation self-efficacy) or job resources (such as colleague support and job autonomy), which effectively buffer the resource-depletion process in the face of violence and allow them to maintain a relatively adaptive psychological state. Conversely, Class 2 healthcare workers may already be caught in a state of resource loss. Violence continuously depletes psychological resources, the progressive depletion of resources intensifies the experience of burnout, and burnout in turn further weakens the individual’s ability to cope effectively with violent incidents, forming a self-reinforcing vicious cycle among these three elements. By revealing the complex joint distribution patterns of workplace violence and burnout, this study extends the boundaries of applying the person-centered research paradigm in this field.

### 4.2 Analysis of the influencing factors of the optimal latent profiles

Work-family conflict was the strongest independent predictor distinguishing the two latent profiles. According to role stress theory, healthcare workers simultaneously occupy the dual roles of medical service provider and family member (58, 59); when the demands of these two roles conflict at the temporal, strain, and behavioral levels and are difficult to reconcile, work-family conflict arises. In the contemporary Chinese medical service environment, healthcare workers commonly face occupational demands such as long working hours, irregular schedules, and frequent night shifts, and these work characteristics systematically encroach on the time and energy necessary to fulfill family roles (60). More critically, the experience of workplace violence may further intensify the accumulation of stress. The anger, fear, and emotional numbness that healthcare workers experience after being subjected to violence may erode their emotional availability and capacity for positive responsiveness in family interactions, leading to a weakening of family support functions (61). This in turn deprives healthcare workers of opportunities to obtain emotional recovery and psychological-resource replenishment from the family domain, leaving them without effective buffering mechanisms when confronting persistent occupational stress, and ultimately significantly raising their probability of belonging to the high-risk profile.

Although the univariate analysis showed statistically significant differences between the two profiles in hospital role, gender, educational level, and monthly income level, none of these demographic and occupational characteristics reached significance in the multivariable regression model. This suggests that the influence of demographic variables on profile membership may operate primarily and indirectly through psychosocial mechanisms such as work-family conflict and emotional labor, rather than directly determining the level of risk. For example, women were more likely to belong to Class 2 in the univariate analysis, but the gender effect disappeared once work-family conflict was entered into the model. This indicates that the gender difference may essentially reflect the higher family-role expectations and the more pronounced work–family role conflict borne by female healthcare workers within the Chinese sociocultural context (62), rather than an independent increase in risk attributable to gender itself. Furthermore, this result underscores that intervention pathways centered on psychosocial factors have a universal applicability that cuts across demographic characteristics. Regardless of a healthcare worker’s gender, education, or income, reducing work-family conflict and optimizing emotion-regulation strategies are core intervention points for improving their occupational mental health.

### 4.3 Practical implications

This study found that more than half of healthcare workers belonged to the high-risk joint profile, and that there was a significant positive correlation between workplace violence and burnout.Therefore, violence prevention should be a core and priority component of hospital occupational health management systems. Specific intervention measures should include the following two aspects for reference. First, hospitals should establish a standardized, non-punitive system for reporting and tracking violent incidents, improving the detection rate of and response speed to violent incidents by reducing victims’ reporting barriers and psychological concerns. Second, hospitals should deploy adequate security personnel in departments with a high incidence of violence and optimize the physical environment, for example by improving lighting in waiting areas, installing emergency call buttons, and installing comprehensive surveillance equipment.

Given that work-family conflict demonstrated an extremely strong independent predictive effect, hospital administrators should incorporate the promotion of work–family balance into the organization’s health strategy. From the practical-translation perspective of Conservation of Resources theory, hospitals should implement flexible scheduling systems and humane shift arrangements, allowing healthcare workers to autonomously choose shifts and adjust workloads within a certain range according to their family caregiving needs, thereby reducing the rigid conflict between working hours and family responsibilities and enhancing their sense of control over the work–family interface. At the same time, for healthcare workers with childcare or elderly-care needs, hospitals should provide on-site childcare services, caregiving leave policies, and relevant social support information resources, substantively alleviating the squeezing effect of family caregiving burdens on the work role. These measures essentially constitute proactive resource investment at the organizational level, helping healthcare workers effectively expand the pool of resources they can draw on to meet the demands of both work and family, and thereby concretely reducing their probability of falling into resource loss and belonging to the high-risk profile.

### 4.4 Limitations and directions for future research

Despite the above findings, this study still has a number of shortcomings and limitations. First, the cross-sectional design of this study precludes rigorous causal inference and prevents the determination of the temporal-sequence direction of the relationships among the variables. For example, this study showed that work-family conflict was the strongest predictor of belonging to the high-risk profile, but from a bidirectional and dynamic perspective, the continued elevation of workplace violence and burnout levels may likewise, in turn, aggravate work-family conflict. In addition, the cross-sectional design cannot capture the developmental trajectories, fluctuation rhythms, and key turning points of the core variables along the temporal dimension, which limits our comprehensive understanding of the dynamic interactive processes among violence, burnout, and work-family conflict.

Second, the sample representativeness and measurement methods of this study have certain limitations that need to be fully considered when generalizing the results. With respect to sample composition, this study was predominantly composed of female nurses from tertiary Class A hospitals and was recruited from a specific geographic region, which means that due caution must be exercised when generalizing the conclusions to primary healthcare institutions, male healthcare worker populations, and other cultural and healthcare-system contexts. Healthcare institutions of different levels and types may differ significantly in the incidence of workplace violence, the distribution of types of violence, and the coping resources available.

With respect to measurement methods, all core variables were measured using self-report questionnaires. Although Harman’s single-factor test suggested that common method bias was within an acceptable range, the potential interference of social desirability effects and recall bias on the study results still cannot be completely ruled out. Future research should consider incorporating multi-source rating data and multi-method measurement strategies to further reduce the potential influence of bias at the methodological level.

Fourth, the primary latent profile model used item-level indicators and, like all mixture models, assumed conditional independence of indicators within class; residual item covariance was not modelled, which may bias class enumeration upwards. Although a parcel-level sensitivity analysis reproduced the two-profile structure, the estimated size of the high-risk profile varied with indicator specification, and replication with alternative parameterisations (e.g., mixture factor models or models allowing within-class residual covariance) is warranted.

Fifth, two of the profile indicators were markedly leptokurtic. Latent profile analysis with continuous indicators assumes within-class normality, and violations of this assumption can inflate the estimated number of classes even under robust estimation. Although our enumeration strategy explicitly discounted the BLRT for this reason and the two-profile structure was reproduced at the parcel level, future work should confirm these profiles using mixture models that explicitly accommodate non-normal within-class distributions or factor mixture models.

Based on the above limitations, this study makes the following recommendations. First, future research should conduct multi-wave longitudinal follow-up studies, employing a cross-lagged panel design or the more advanced random-intercept cross-lagged panel model, in order to effectively separate stable between-person differences from within-person dynamic changes and to precisely test the dominant causal direction, the temporal lag structure of effects, and the potential bidirectional reciprocal relationships among workplace violence, burnout, and work-family conflict. Second, future research should incorporate organizational-level contextual variables—such as departmental safety climate, head nurses’ leadership style, team psychological safety, and perceived organizational justice—and use multilevel analysis models to examine the cross-level interaction effects of individual- and organizational-level factors, thereby constructing a more comprehensive and ecologically valid multilevel integrated model of risk and protective factors. Third, based on the characteristics of the high-risk profile and its core influencing factors, future research should adopt a mixed-methods research paradigm to develop and rigorously validate targeted composite intervention programs—for example, multi-module interventions integrating violence-prevention training, cognitive restructuring of work-family conflict, and deep-acting skill development—and to test their actual effectiveness in improving occupational mental health through multicenter randomized controlled trials. In addition, using qualitative research methods to explore in depth the psychological adaptation processes and resource-mobilization strategies of healthcare workers after experiencing violence would help enrich, at the micro level, the theoretical understanding of the mechanisms underlying the formation of the joint profiles.

## Data Availability

The raw data supporting the conclusions of this article will be made available by the authors, without undue reservation.

## References

[1] Abreu AR Gonçalves F Oliveira S Ribeiro I . Workplace violence against healthcare workers: a scoping review of reporting practices, barriers to reporting and institutional responses (2020–2025). BMC Health Serv Res. (2026) 26(1): 419. doi: 10.1007/978-3-031-25984-5_870 PMC13032240 41723495

[2] Çakır O Akkoç İ Arun K Dığrak E Uluırmak Ünlüeroğlugil HS . The complex dynamics of violence and burnout in healthcare: a closer look at physicians and nurses. Int Nurs Rev. (2025) 72:e70075. doi: 10.1111/inr.70075 PMC12272327 40678941

[3] de Oliveira AC de Souza AS Marcheti PM Sperotto MM Sarti ECFB de Andrade Porto JV . Occupational violence in health care: a systematic review. Acta Psychologica. (2025) 260:105611. doi: 10.1016/j.actpsy.2025.105611 41066860

[4] Feruglio L Bressan V Cadorin L . Violence against nurses during care: a systematic review. J Clin Nurs. (2025) 34:1106–23. doi: 10.1111/jocn.17424 39350468

[5] Guo X Cui M Lu Z Huang Z Wang Y . The impact of workplace violence on nursing staff's physical and mental health: a cross‐sectional survey at a teaching hospital. J Advanced Nurs. (2025) 1–12. doi: 10.1111/jan.70261 41439330

[6] Cannizzaro D Saguatti I Caleffi D Rovesti S Ferri P . Physical and psychological consequences for nurses affected by workplace violence: a scoping review. BMC Nurs. (2025) 25(1): 40. doi: 10.1186/s12912-025-04182-7 41372965 PMC12802330

[7] Morales Palomares S Gheri F Ramacciati N . Cultural dimensions of workplace violence in healthcare: a narrative review. J Nurs Manage. (2025) 2025:8336854. doi: 10.1155/jonm/8336854 41438995 PMC12721393

[8] Korkis L Murphy T O’Rourke MW . Workplace violence as a crisis: using O’Rourke’s theory as a framework to foster role clarity and professional role commitment. Nurs Outlook. (2025) 73:102391. doi: 10.1016/j.outlook.2025.102391 40199172

[9] Lyver B Singh B Balzer N Agnihotri M Hulme J Chan K . (2025). Exploring the impact of workplace violence in urban emergency departments: a qualitative study. Healthcare (Basel). 10.3390/healthcare13060679 PMC11941988 40150529

[10] Edú-Valsania S Laguía A Moriano JA . Burnout: a review of theory and measurement. Int J Environ Res Public Health. (2022) 19:1780. doi: 10.3390/ijerph19031780 35162802 PMC8834764

[11] Sharifi M Asadi-Pooya AA Mousavi-Roknabadi RS . Burnout among healthcare providers of COVID-19; a systematic review of epidemiology and recommendations. Arch Acad Emergency Med. (2020) 9:e7. doi: 10.22037/aaem.v9i1.1004 PMC7812159 33490964

[12] Getie A Ayenew T Amlak BT Gedfew M Edmealem A Kebede WM . Global prevalence and contributing factors of nurse burnout: an umbrella review of systematic review and meta-analysis. BMC Nurs. (2025) 24:596. doi: 10.1186/s12912-025-03266-8 40420259 PMC12108038

[13] Batanda I . Prevalence of burnout among healthcare professionals: a survey at fort portal regional referral hospital. NPJ Ment Health Res. (2024) 3:16. doi: 10.1038/s44184-024-00061-2 38710834 PMC11074248

[14] Holmér S Nedlund A-C Thomas K Krevers B . How health care professionals handle limited resources in primary care–an interview study. BMC Health Serv Res. (2023) 23:6. doi: 10.1186/s12913-022-08996-y PMC9808951 36597086

[15] Zheng Y Wang X Yang L . Explore the correlations of occupational commitment, psychological resilience, job satisfaction, and burnout in kindergarten teachers’ turnover intentions in rural China. Front Psychol. (2025) 16:1605831. doi: 10.3389/fpsyg.2025.1605831 40657573 PMC12247534

[16] Shi L Xin S Li D-N Song P-Y Tian Y Ren F . The mediating role of resilience in job satisfaction and burnout among Chinese plateau military personnel. Sci Rep. (2025) 15:13785. doi: 10.1038/s41598-025-97673-1 40258917 PMC12012146

[17] Alzoubi MM Al-Mugheed K Oweidat I Alrahbeni T Alnaeem MM Alabdullah AAS . Moderating role of relationships between workloads, job burnout, turnover intention, and healthcare quality among nurses. BMC Psychol. (2024) 12:495. doi: 10.1186/s40359-024-01891-7 39300567 PMC11414086

[18] Ahmed AK Atta MHR El-Monshed AH Mohamed AI . The effect of toxic leadership on workplace deviance: the mediating effect of emotional exhaustion, and the moderating effect of organizational cynicism. BMC Nurs. (2024) 23:669. doi: 10.1186/s12912-024-02308-x 39300476 PMC11414162

[19] Wang X Li C Chen Y Zheng C Zhang F Huang Y . Relationships between job satisfaction, organizational commitment, burnout and job performance of healthcare professionals in a district-level health care system of Shenzhen, China. Front Psychol. (2022) 13:992258. doi: 10.3389/fpsyg.2022.992258 36518969 PMC9742526

[20] Deng L Yang D Liang G Hu C Zhang P . The impact of generative AI'S information delivery methods on emotional exhaustion among bullying roles in the medical workplace. Front Public Health. (2025) 13:1649342. doi: 10.3389/fpubh.2025.1724549 41030366 PMC12477917

[21] Christensen SS Wilson BL Cummins MR Eaton J Iacob E Hansen SD . Exploring nurses' emotional reactions to and reporting of patient-on-nurse workplace violence: a mixed-methods study. Int J Nurs Stud. (2024) 153:104724. doi: 10.1016/j.ijnurstu.2024.104724 38437757

[22] Kafle S Paudel S Thapaliya A Acharya R . Workplace violence against nurses: a narrative review. J Clin Trans Res. (2022) 8:421. doi: 10.18053/jctres.08.202205.010 36212701 PMC9536186

[23] Bakker AB Demerouti E . The job demands‐resources model: state of the art. J Managerial Psychol. (2007) 22:309–28. doi: 10.1108/0268394071073311535579975

[24] Zeng Z Xie G He Y Zhou S . Exploring psychological pathways between workplace violence and burnout among nurses in Chinese tertiary hospitals. Sci Rep. (2025) 15:35695. doi: 10.1038/s41598-025-19671-7 41083622 PMC12518848

[25] Cao Y Gao L Fan L Jiao M Li Y Ma Y . The influence of emotional intelligence on job burnout of healthcare workers and mediating role of workplace violence: a cross sectional study. Front Public Health. (2022) 10:892421. doi: 10.3389/fpubh.2022.892421 35646806 PMC9130825

[26] Tsukamoto SAS Galdino MJQ Barreto MFC Martins JT . Burnout syndrome and workplace violence among nursing staff: a cross-sectional study. Sao Paulo Med J. (2022) 140:101–7. doi: 10.1590/1516-3180.2021.0068.r1.31052021 34932780 PMC9623842

[27] French KA Allen TD Kidwell KE . When does work-family conflict occur? J Vocational Behav. (2022) 136:103727. doi: 10.1016/j.jvb.2022.10372742574925

[28] Hetrick AL Haynes NJ Clark MA Sanders KN . The theoretical and empirical utility of dimension-based work–family conflict: a meta-analysis. J Appl Psychol. (2024) 109:987. doi: 10.1037/apl0000552 37289526

[29] Lu Y Han Y Wang Y Tang Y . Understanding the impact of family-to-work conflict and enrichment on the performance of entrepreneurial firms: a conservation of resources perspective. Int J Hum Resource Manage. (2023) 34:1097–131. doi: 10.1080/09585192.2021.197624437339054

[30] Park HI Jang J Nam JS . Physical activity buffers the effects of work-family conflict on work engagement through mastery recovery experience. Curr Psychol. (2023) 42:348–58. doi: 10.1007/s12144-021-01463-730311153

[31] Pisaniello SL Winefield HR Delfabbro PH . The influence of emotional labour and emotional work on the occupational health and wellbeing of South Australian hospital nurses. J Vocational Behav. (2012) 80:579–91. doi: 10.1016/j.jvb.2012.01.01542574925

[32] Bokek-Cohen Y Tarabeih M . (2021). The emotional labor of the transplant coordinator: an inherent predicament. Transplant Proc. 53(6):1846–52. doi: 10.1016/j.transproceed.2021.06.022 34247860

[33] Humphrey RH Ashforth BE Diefendorff JM . The bright side of emotional labor. J Organizational Behav. (2015) 36:749–69. doi: 10.1002/job.201942587375

[34] Grandey AA . When “the show must go on”: surface acting and deep acting as determinants of emotional exhaustion and peer-rated service delivery. Acad Manage J. (2003) 46:86–96. doi: 10.2307/30040678

[35] Zhang F . Surface vs. deep acting in EFL instruction: mediating and moderating roles of emotion regulation and intelligence in burnout. Front Psychol. (2025) 16:1611941. doi: 10.3389/fpsyg.2025.1611941 41312265 PMC12646906

[36] Christiansen DM McCarthy MM Seeman MV . Where sex meets gender: how sex and gender come together to cause sex differences in mental illness. Front Psychiatry. (2022) 13:856436. doi: 10.3389/fpsyt.2022.856436 35836659 PMC9273892

[37] Street AE Dardis CM . Using a social construction of gender lens to understand gender differences in posttraumatic stress disorder. Clin Psychol Rev. (2018) 66:97–105. doi: 10.1016/j.cpr.2018.03.001 29580673

[38] Sever M Tiryaki A . Emotional labor, conflicting caregiving responsibilities and resilience among foreign female caregivers in Japan: a photovoice study. Gender Work Organ. (2025) 32:949–76. doi: 10.1111/gwao.1319540046247

[39] Zhu H Yang X Xie S Zhou J . Prevalence of burnout and mental health problems among medical staff during the COVID-19 pandemic: a systematic review and meta-analysis. BMJ Open. (2023) 13:e061945. doi: 10.1136/bmjopen-2022-061945 37474193 PMC10360428

[40] Paneni V Gambelunghe C Tomassini L Buresti G Rondinone BM Persechino B . (2025). Correlations between gender, age, and occupational factors on the work ability index among healthcare professionals. Healthcare (Basel). 13(7):702. doi: 10.3390/healthcare13070702 PMC11988494 40218000

[41] Tein J-Y Coxe S Cham H . Statistical power to detect the correct number of classes in latent profile analysis. Struct Equation Modeling: A Multidiscip J. (2013) 20:640–57. doi: 10.1080/10705511.2013.824781 24489457 PMC3904803

[42] Peduzzi P Concato J Kemper E Holford TR Feinstein AR . A simulation study of the number of events per variable in logistic regression analysis. J Clin Epidemiol. (1996) 49:1373–9. doi: 10.1016/s0895-4356(96)00236-3 8970487

[43] Wang P Wang M Hu G Wang Z . Study on the relationship between workplace violence and work ability among health care professionals in Shangqiu City. Wei Sheng Yan Jiu= J Hygiene Res. (2006) 35:472–4. 16986527

[44] Tian Y Yue Y Wang J Luo T Li Y Zhou J . Workplace violence against hospital healthcare workers in China: a national WeChat-based survey. BMC Public Health. (2020) 20:582. doi: 10.1186/s12889-020-08708-3 32349727 PMC7189471

[45] Xu H Yuan Y Gong W Zhang J Liu X Zhu P . Reliability and validity of the Chinese version of Oldenburg Burnout Inventory for Chinese nurses. Nurs Open. (2022) 9:320–8. doi: 10.1002/nop2.1065 34546665 PMC8685855

[46] Zhang S Xiao K Tian Z . Burnout and personality profiles among Chinese nurses. Behav Sci. (2024) 14:1117. doi: 10.3390/bs14121117 39767258 PMC11674007

[47] Huang Z Huang F Liang Q Liao F Tang C Luo M . Socioeconomic factors, perceived stress, and social support effect on neonatal nurse burnout in China: a cross-sectional study. BMC Nurs. (2023) 22:218. doi: 10.1186/s12912-023-01380-z 37357312 PMC10291763

[48] Carlson DS Kacmar KM Williams LJ . Construction and initial validation of a multidimensional measure of work–family conflict. J Vocational Behav. (2000) 56:249–76. doi: 10.1006/jvbe.1999.171321632986

[49] Jung N Kim M . Assessing work-family conflict experienced by Chinese parents of young children: validation of the Chinese version of the work and family conflict scale. Child Psychiatry Hum Dev. (2023) 54:123–33. doi: 10.1007/s10578-021-01236-0 34415459

[50] Yang C Chen Y Zhao X . Emotional labor: scale development and validation in the Chinese context. Front Psychol. (2019) 10:2095. doi: 10.3389/fpsyg.2019.02095 31620048 PMC6759872

[51] Huang G Zheng Y Shen G Wang H Wang L . The dual pathways of emotional labor in psychiatric nursing: the adaptive and maladaptive emotional strategies. Front Psychiatry. (2026) 17:1719188. doi: 10.3389/fpsyt.2026.1719188 41947809 PMC13051302

[52] Kline RB . Principles and Practice of Structural Equation Modeling. New York, NY: Guilford publications. (2023).

[53] Bauer DJ Curran PJ . Distributional assumptions of growth mixture models: implications for overextraction of latent trajectory classes. psychol Methods. (2003) 8:338. doi: 10.4324/9780367855222-2 14596495

[54] Zhou S Zeng Z . Latent profiles and associated factors of workplace violence among nurses in tertiary hospitals: a cross-sectional study. Front Psychiatry. (2025) 16:1651237. doi: 10.3389/fpsyt.2025.1651237 41019593 PMC12460260

[55] Li X Yang F Chen X Zhao H Yu R . Latent profile analysis of nurse burnout categories and influencing factor differences among nurses in Southwest China. Front Public Health. (2026) 14:1764970. doi: 10.3389/fpubh.2026.1764970 41948020 PMC13050854

[56] Baka L Prusik M Jasielska D . Toward a better understanding of the health impairment process. Types of demand and burnout component matter. Front Psychiatry. (2023) 13:1037053. doi: 10.3389/fpsyt.2022.1037053 36699490 PMC9868600

[57] Demerouti E . Job demands-resources and conservation of resources theories: How do they help to explain employee well-being and future job design? J Business Res. (2025) 192:115296. doi: 10.1016/j.jbusres.2025.11529642574925

[58] NorDenmark M . Multiple social roles and well-being: A longitudinal test of the role stress theory and the role expansion theory. Acta Sociologica. (2004) 47:115–26. doi: 10.1177/0001699304043823

[59] Boumans NP Dorant E . Double‐duty caregivers: healthcare professionals juggling employment and informal caregiving. A survey on personal health and work experiences. J Advanced Nurs. (2014) 70:1604–15. doi: 10.1111/jan.12320 24289650

[60] Poissonnet CM Véron M . Health effects of work schedules in healthcare professions. J Clin Nurs. (2000) 9:13–23. doi: 10.1046/j.1365-2702.2000.00321.x 11022488

[61] Chan S Khong P Wang W . Psychological responses, coping and supporting needs of healthcare professionals as second victims. Int Nurs Rev. (2017) 64:242–62. doi: 10.1111/inr.12317 27679402

[62] Diao D Chen X Zhong L Zhang H Zhang J . Sex differences in burnout and work-family conflict among Chinese emergency nurses: a cross-sectional study. Front Public Health. (2024) 12:1492662. doi: 10.3389/fpubh.2024.1492662 39712298 PMC11659251

